# Beyond ventriculo-arterial coupling: can arterial waveform-derived profiling refine prediction of post-induction hypotension?

**DOI:** 10.1186/s40635-026-00932-2

**Published:** 2026-08-04

**Authors:** Alessandra Ciccozzi, Chiara Angeletti

**Affiliations:** 1 Department of Anesthesia and Intensive Care, San Salvatore University Hospital, L’Aquila, Italy; 2 Department of Life, Health and Environmental Sciences-University of L’Aquila (MESVA), L’Aquila, Italy; 3 Department of Anesthesia, Intensive Care and Pain Therapy, Hospital “Giuseppe Mazzini”, Piazza Italia, 64100 Teramo Italy

## Dear editor-in-chief,

We read with great interest the prospective observational cohort study by *Le et al.*, which evaluated the predictive value of ventriculo-arterial coupling (VAC) for post-induction hypotension (PIH) in patients undergoing elective non-cardiac surgery [1]. The study makes an important physiological contribution by showing that impaired VAC, expressed as an Ea/Ees ratio > 1, was independently associated with PIH and improved risk reclassification when added to clinical and echocardiographic variables. These findings support the concept that PIH is not merely a pressure-threshold phenomenon or an isolated pharmacological consequence of anaesthetic induction but may reflect a pre-existing mismatch between ventricular performance and arterial load.

The work by *Le et al.*. also raises a relevant question for future research: can pre-induction cardiovascular vulnerability be captured using continuous arterial waveform-derived variables immediately before induction?

Echocardiography-derived Ea/Ees offers a robust physiological framework, but its implementation requires transthoracic echocardiography, non-invasive blood pressure measurement, and estimates of effective arterial elastance and end-systolic elastance. In contrast, pulse contour and pressure-recording analytical methods may provide continuous, minimally invasive information for patients who already require invasive arterial monitoring for clinical reasons.

This question is supported by the work of *Aktas Yildirim et al.*, who reported that pre-induction arterial elastance, measured with MostCare/PRAM, was associated with hypotension after induction of anaesthesia [2].

In a related PRAM-monitored study, Aktas Yildirim and colleagues also suggested that stroke volume index and cardiac power output may contribute to post-induction hemodynamic risk [3].

Taken together, these findings suggest that arterial load, flow generation, and cardiovascular power may all contribute to susceptibility to PIH. However, the available evidence remains fragmented, as most studies have evaluated single predictors or limited sets of variables in relatively small cohorts.

To date, no prospective study has integrated VAC-informed concepts with arterial waveform-derived indices of arterial load, cardiac performance, cardiovascular efficiency, and preload sensitivity in the immediate pre-induction window.

The need for an integrated approach is further supported by the literature on endotypes of intraoperative hypotension. *Kouz et al.* used hierarchical clustering in patients undergoing major abdominal surgery who were monitored with invasive pulse wave analysis and identified six distinct hypotension endotypes: myocardial depression, bradycardia, vasodilation with an increased cardiac index, vasodilation without an increased cardiac index, hypovolemia, and mixed type [4].

More recently, *Zhu et al.* applied a temporal deep learning approach to high-resolution intraoperative hemodynamic data and identified five hypotension endotypes, suggesting that hypotensive episodes may evolve dynamically over time [5].

These studies reinforce the view that similar reductions in arterial pressure may arise from different hemodynamic mechanisms.

However, these studies primarily classified hypotension after it had already occurred. The immediate pre-induction period remains underexplored. This distinction is clinically relevant because anaesthesia induction represents a short, relatively standardized window before later intraoperative confounders emerge, including bleeding, pneumoperitoneum, positional changes, neuraxial analgesia, cumulative fluid administration, and prolonged vasopressor exposure.

Future prospective studies should therefore assess whether pre-induction hemodynamic vulnerability profiles can be identified before the onset of PIH. Such studies could enroll adult patients undergoing elective major noncardiac surgery for whom invasive arterial monitoring is already indicated as part of routine care. Baseline variables obtained immediately before induction could include arterial pressure, heart rate, stroke volume, stroke volume index, cardiac output, cardiac index, systemic vascular resistance, systemic vascular resistance index, arterial elastance, dynamic arterial elastance, cardiac power output, cardiac cycle efficiency, dP/dt max, stroke volume variation, and pulse pressure variation.

Rather than aiming immediately to validate a definitive prediction model, these studies should first be framed as hypothesis-generating. Their purpose would be to determine whether distinct pre-induction profiles, such as high arterial load, reduced cardiovascular performance, preload-sensitive, compensated/low-risk, or mixed profiles, are associated with the incidence, timing, severity, and treatment requirements of early PIH. A standardized endpoint, such as MAP < 65 mmHg for at least 1 min, would improve comparability with the broader PIH literature, whereas relative definitions, such as a MAP decrease > 30% from baseline and/or systolic arterial pressure < 90 mmHg, may capture clinically relevant, patient-specific hypotension.

This physiology-driven approach may complement the VAC framework proposed by *Le et al.* Echocardiography-derived Ea/Ees provides a structured estimate of ventriculo-arterial interaction before induction, whereas arterial waveform-derived profiling may offer continuous, immediate peri-induction information on arterial load, cardiac performance, cardiovascular efficiency, and preload sensitivity. These strategies should therefore be viewed as complementary rather than competing approaches to perioperative hemodynamic risk stratification.

In conclusion, *Le et al.* provide compelling evidence that impaired ventriculo-arterial coupling is associated with PIH. Future studies should determine whether integrated pre-induction hemodynamic profiles derived from advanced arterial waveform analysis can further characterize individual vulnerability to PIH and support more mechanism-oriented perioperative blood pressure management.

## Data Availability

Data sharing is not applicable to this article as no datasets were generated or analysed.

## Abbreviations

CCE: Cardiac cycle efficiency
CI: Cardiac index
CO: Cardiac output
CPO: Cardiac power output
Ea: Effective arterial elastance
Eadyn: Dynamic arterial elastance
Ea/Ees: Ventriculo-arterial coupling ratio
Ees: End-systolic elastance
MAP: Mean arterial pressure
PIH: Post-induction hypotension
PPV: Pulse pressure variation
PRAM: Pressure recording analytical method
SAP: Systolic arterial pressure
SV: Stroke volume
SVI: Stroke volume index
SVR: Systemic vascular resistance
SVRI: Systemic vascular resistance index
SVV: Stroke volume variation
VAC: Ventriculo-arterial coupling

## References

[1] LHC, TCBH, NTTT et al (2026) Predictive value of ventriculo-arterial coupling for hypotension after induction of anaesthesia: a prospective observational cohort study. Intensive Care Med Exp 14(1):50. 10.1186/s40635-026-00899-0. Published 2026 Apr 21 10.1186/s40635-026-00899-0 PMC13100084 42012621

[2] Aktas Yildirim S, Sarikaya ZT, Dogan L, Ulugol H, Gucyetmez B, Toraman F (2023) Arterial Elastance: A Predictor of Hypotension Due to Anesthesia Induction. J Clin Med 12(9):3155 Published 2023 Apr 27. 10.3390/jcm12093155 37176595 10.3390/jcm12093155 PMC10179039

[3] Yildirim SA, Dogan L, Sarikaya ZT, Ulugol H, Gucyetmez B, Toraman F (2023) Hypotension after Anesthesia Induction: Target-Controlled Infusion Versus Manual Anesthesia Induction of Propofol. J Clin Med 12(16):5280 Published 2023 Aug 14. 10.3390/jcm12165280 37629322 10.3390/jcm12165280 PMC10455971

[4] Kouz K, Brockmann L, Timmermann LM et al (2023) Endotypes of intraoperative hypotension during major abdominal surgery: a retrospective machine learning analysis of an observational cohort study. Br J Anaesth 130(3):253–261. 10.1016/j.bja.2022.07.056 36526483 10.1016/j.bja.2022.07.056

[5] Zhu Y, Hao X, Li P et al (2025) Identification of intraoperative hypotension endotypes and revolution with a temporal deep learning algorithm. Perioper Med (Lond) 14(1):109. 10.1186/s13741-025-00598-6. Published 2025 Oct 14 41088430 10.1186/s13741-025-00598-6 PMC12523014

